# Precocious endothelial dysfunction in patients with congenital generalized lipodystrophy (Berardinelli-Seip syndrome) evaluated by two different methods

**DOI:** 10.1186/1758-5996-7-S1-A111

**Published:** 2015-11-11

**Authors:** Virgínia Oliveira Fernandes, Ana Paula Dias Rangel Montenegro, Clarisse Mourão Melo Ponte, Lia Beatriz de Azevedo Souza Karbage, Manuela Montenegro Dias de Carvalho, Daniel Duarte Gadelha, Synara Cavalcante Lopes, Marivaldo Loyola Aragão, Ana Paula Abreu Martins Sales, Cristiane Bezerra Rocha Liberato, Ana Gardênia Liberato Ponte Farias, Catarina Brasil D'Alva, Francisco Herlânio Costa Carvalho, Izabella Tamira Galdino Farias Vasconcelos, Carla Antoniana Ferreira de Almeida Vieira, Ana Paula Germano Lopes Cavalcante, Mariella Zaiden Rezende Reis, Renan Magalhães Montenegro

**Affiliations:** 1 Faculdade de Medicina da Universidade Federal do Ceará, Fortaleza, Brazil

## Background

Berardinelli-Seip syndrome (BS) is a rare disease characterized by severe insulin resistance and absence of subcutaneous fat since birth or early childhood. This condition Results in lipids's ectopic deposit (muscle, liver and arterial walls), which explains its clinical complications as diabetes, hepatic injury, hyperlipidemia and premature atherosclerosis.

## Objective

To evaluate endothelial function with the flow-mediated dilatation of the brachial artery (FMD) and peripheral arterial tonometry (ENDOPAT) in patients with BS.

## Matrials and methods

A cross-sectional study with 11 patients with BS and 14 healthy individuals. They performed clinical evaluation, laboratory exams and FMD evaluation. The BS group also performed EndoPAT evaluation. The data were analyzed in STATA 11.2.

## Results

There was no difference in age and gender between the groups. After adjusted for sex, age and height, BS group had high blood pressure in 88.89%; hypertriglyceridemia in 69.23%; low HDL-c in 84.62% and hypercholesterolemia in 25% of the subjects. Left ventricular hypertrophy was observed in 40% of the BS group and 50% of BS patients had diabetes. Comparing BS group with control group were observed, respectively, mean systolic BP (mmHg): 127±23.63 vs 102.21±13.40 (p=0.002), diastolic BP (mmHg): 78.11±16.48 vs 63.21±5.23 (p=0.002), BMI (kg/m^2^): 18.94±2.50 vs 19.93±3.27 (p=0.379), the body fat percentage at bioelectrical impedance analysis (%): 8.56±3.53 vs 24.5±7.67 (p=0.000), fasting plasma glucose (mg/dL): 109.14±78.60 vs 82.43±10.87 (p=0.713), total cholesterol (mg/dL): 192.46±151.64 vs 30.33±179.83 (p=0.423), HDL-c (mg/dL): 29.84±8.73 vs 42.14±11.45 (p=0.004), LDL-c (mg/dL): 80.82±28.04 vs. 90.21±23.29 mg/dL (p=0.125), triglycerides (mg/dL): 188.08±186.09 vs 80.57±38.39 (p=0.003). Endothelial disfunction by FMD was presented in 81.8% of the BS group vs. 30.77% in control group (p=0.012). The prevalence ratio was 2.86 (confidence interval: 1.19-6.86). Endothelial disfunction by EndoPAT was observed in 50% of the subjects with a natural logarithm reactive hyperemia index (LnRHI index) of 0.49±0.15. There was a mild agreement between the diagnosis of endothelial dysfunction assessed by FMD and EndoPAT (kappa: 0.40, p=0.056).

## Conclusion

The results showed that patients with BS presented endothelial dysfunction, even in early ages. It shows the necessity of early intervention in patients to avoid cardiovascular outcomes.

